# Impact of health literacy and social support on medication adherence in patients with hypertension: a cross-sectional community-based study

**DOI:** 10.1186/s12872-023-03117-x

**Published:** 2023-02-19

**Authors:** Aizhen Guo, Hua Jin, Jianbo Mao, Weihong Zhu, Ye Zhou, Xuhua Ge, Dehua Yu

**Affiliations:** 1 Department of General Practice, Yangpu Hospital, School of Medicine, Tongji University, Shanghai, 200090 China; 2 Shanghai General Practice and Community Health Development Research Center, Shanghai, 200090 China; 3 Changbai Community Health Service Center, Yangpu District, Shanghai, 200093 China; 4 Wujiaochang Community Health Service Center, Yangpu District, Shanghai, 200433 China; 5 Yanji Community Health Service Center, Yangpu District, Shanghai, 200093 China

**Keywords:** Medication adherence, Educational level, Social support, Health literacy, Hypertension, Structural equation model

## Abstract

**Background:**

Previous studies have examined the associations of health literacy and social support with medication adherence among patients with hypertension. However, limited evidence exists regarding the mechanisms underlying the relationship between these factors and medication adherence.

**Purpose:**

To explore the prevalence of medication adherence and its determinants in patients with hypertension in Shanghai.

**Methods:**

A community-based cross-sectional study was conducted among 1697 participants with hypertension. We collected sociodemographic and clinical characteristics as well as data regarding health literacy, social support, and medication adherence using questionnaires. We examined interactions among the factors using a structural equation model.

**Results:**

The participants included 654 (38.54%) patients with a low degree of medication adherence and 1043 (61.46%) patients with a medium/high degree of adherence. Social support directly influenced adherence (*β* = 0.165, *P* < 0.001) and indirectly influenced adherence through health literacy (*β* = 0.087, *P* < 0.001). Health literacy directly influenced adherence (*β* = 0.291, *P* < 0.001). Education indirectly affected adherence through both social support (*β* = 0.048, *P* < 0.001) and health literacy (*β* = 0.080, *P* < 0.001). Moreover, there was a sequential mediating effect of social support and health literacy on the association between education and adherence (*β* = 0.025, *P* < 0.001). After controlling for age and marital status, similar results were also obtained, indicating a good model fit.

**Conclusions:**

The degree of medication adherence among hypertensive patients needs to improve. Health literacy and social support had both direct and indirect effects on adherence, and thus, these factors should be considered as tools to improve adherence.

**Supplementary Information:**

The online version contains supplementary material available at 10.1186/s12872-023-03117-x.

## Background

Hypertension is one of the most common chronic conditions and a leading cause of cardiovascular and all-cause mortality worldwide. By 2025, ~ 29% of the global population is expected to have this disease [1]. In China, there are 270 million patients with hypertension, and this disease has become the primary contributor to disability-adjusted life years, resulting in 24.6% of deaths [2]. The prevention and treatment of hypertension remain important priorities in the field of public health [3, 4]. Medical care is recommended for most patients when lifestyle changes can no longer successfully control blood pressure. The mortality risk of hypertension and its complications can be effectively controlled with low-cost off-patent drugs. However, 30–50% of medications are not taken as prescribed among adult patients [5–7]. Medication nonadherence in patients with hypertension has been one of the biggest barriers to the optimal control of blood pressure, the prevention of multiple complications, and the reduction of health care costs.

Understanding the factors that influence medication adherence is critical for improving medication adherence. The factors that are known to be related to medication adherence have been classified into five categories by the World Health Organization: patient-related factors, socioeconomic influences, health system factors, therapy factors, and condition-related factors. The factors of social support and health literacy among individuals have been clinically underexamined; however, they have recently been recognized to be particularly important for improving adherence. Social support is defined as the "degree to which individuals have the potential access to receive actual or perceived support from his or her social network, such as familial relations, friends, neighbors, colleagues, fellow patients to reduce psychological stress response, relieve mental stress, and improve social adaptability". Low social support has been shown to be associated with uncontrolled blood measures [8]. Previous studies [9–11] have clearly supported that social support is associated with medication adherence and is a critical target for the improvement of medication adherence among patients with hypertension. Improvements in medication adherence among heart failure patients have also been observed when family members provide social support. Health literacy is defined as the "degree to which individuals have the capacity to obtain, process, and understand basic health information and services needed to make appropriate health decisions" [12]. A lower level of health literacy is strongly associated with poor medication adherence [13]. Moreover, a lower level of health literacy has also been shown to be associated with hypertension management [14], disease knowledge [15] and health-related quality of life [16, 17] in studies from different countries. The associations of medication adherence with social support and health literacy in hypertension [18] have been investigated in previous studies; however, the combined effects of these factors on medication adherence and the underlying mechanisms of those relationships in hypertension remain unclear.

**Fig. 1:**
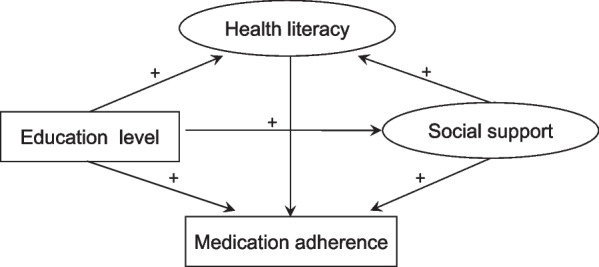
The theoretical hypotheses. Education level has a direct positive relationship with medication adherence, social support and health literacy. Social support has a direct positive relationship with medication adherence and health literacy. Health literacy has a positive relationship with medication adherence. The relationship between education level and medication adherence is mediated by social support and health literacy. The relationship between medication adherence and social support is mediated by health literacy. Circles indicate latent variables. Rectangles indicate exogenous variables

Structural equation modeling (SEM) is a useful analytic technique to investigate the interrelationships between latent variables, such as social support and health literacy, which cannot be measured directly. Additionally, SEM can be used to examine the mediating effect by path analysis. In the current research, we constructed a structural equation model to explore the interactions between medication adherence and education level, social support and health literacy. We also used SEM to examine whether the associations among these factors are mediated by health literacy and/or social support in patients with hypertension. Figure 1 outlines the hypotheses we proposed according to previous studies [9–11, 13, 18, 19]. We hope that the model will reveal relationships between the tested factors and medication adherence that will be helpful for developing effective measures to improve medication adherence among patients with hypertension.

## Methods

### Design

We used a community-based cross-sectional design to analyze the experiences of patients with hypertension in Shanghai regarding prescribed medication adherence.

### Participants

The participants were enrolled from 3 community service centers in Yangpu District in Shanghai, China, from October to December 2021. The inclusion criteria were as follows: (1) diagnosed with hypertension for at least 2 years according to the criteria of the 10th revision of the International Classification of Diseases (ICD-10), (2) aged more than 50 years, (3) currently receiving anti-hypertension therapy, and (4) conscious and voluntary participation. The exclusion criteria were as follows: patients whose condition was too severe to complete the survey (such as dyspnea, dizziness, and palpitation); patients with a history of psychiatric illness; and patients taking antidepressant and/or psychotropic drugs. The institutional review boards in our hospital approved the study protocol, and all participants provided written informed consent.

### Data collection

A total of 1697 patients with hypertension were recruited, and their sociodemographic data (such as age, sex, marital status, education level, monthly income and living status), lifestyle data (such as use of tobacco and alcohol, vegetable consumption and physical activity), and clinical data (such as body mass index (BMI), duration of hypertension, number of medicines taken, blood pressure and complications) were collected via face-to-face interviews. Social support, health literacy and medication adherence were assessed via self-report questionnaires with the help of trained health professionals. Information about serum biochemical markers such as blood glucose and HbA1c was collected from the latest medical records.

### Questionnaires

Adherence to prescription drugs for hypertension was assessed using the eight-item Morisky Medication Adherence Scale (MMAS-8), which has been validated in the Chinese language [20–22]. The MMAS-8 is a structured, self-reported measure of medication adherence among patients. The scale contains seven yes or no items and one five-point scale for specific behavior related to medication adherence. Each question has a score ranging from 0 to 1, and total score of the scale ranges from 0 to 8. The Cronbach's alpha of this scale was 0.739 in this study. As in previous studies, a maximum score of 8 represents a high degree of adherence, a score of 6–8 represents a medium degree of adherence, and a score < 6 represents a low degree of adherence [23].

The social support of patients was evaluated by the social support rate scale (SSRS), which was validated in Chinese by Xiao Shuiyuan and used in a previous study [24]. The ten items of the scale were classified into 3 dimensions: objective support, subjective support and support utilization. Objective support was defined as direct material assistance from social networks or participation in communities; subjective support was defined as the emotional experience and satisfaction of individuals being respected, supported and understood in society; and support utilization mainly refers to whether an individual has access to and receives various types of support and whether individuals attempt to seek support from family, relatives, friends, colleagues, and larger communities. The Cronbach's alpha of the SSRS was 0.711 in this study.

Health literacy was measured using the Health Literacy Management Scale (HeLMS) developed by Jordan et al. [25], which has been validated in the Chinese language [26, 27]. Five items were deleted from the original scale due to similar content between items and not meeting the item selection criteria. The final scale contains 24 items across four dimensions: information acquisition ability (9 items), communication interaction ability (9 items), health improvement willingness (4 items) and economic support willingness (2 items). The items were rated on a five-point Likert scale (from 1 to 5). Higher scores indicate better health literacy. The Cronbach's alpha of the HeLMS scale was 0.977 in this study.

### Statistical analysis

For continuous data, variables were described as the means ± standard deviations (SDs), and the differences among levels of medication adherence (low, medium and high) were compared with analysis of variance (ANOVA). For categorical data, variables were described as counts and percentages, and the differences among the 3 levels of medication adherence were compared using the Mann‒Whitney U or Kruskal‒Wallis test. Spearman correlations were calculated to determine the associations between age, marital status, education, social support, health literacy and medication adherence. A structural equation model (SEM) with maximum likelihood estimation in the lavaan package was used to test the hypotheses outlined in the conceptual model (Fig. 1). To estimate the fit model more stably and to test the significance of indirect effects, a nonparametric bootstrapping method with 5000 samples was used. The bias-corrected 95% confidence interval (CI) and percentile 95% CI were also calculated, and a result was considered significant if the 95% CI did not include zero. The incremental fit index (IFI), comparative fit index (CFI), normed fit index (NFI) ≥ 0.90, and root mean squared error of approximation (RMSEA) ≤ 0.08 were used to confirm the model fit. All analyses were conducted by using SPSS 26.2 and R4.0 software, and a p value of < 0.05 was considered statistically significant.

## Results

### Characteristics among the patients with hypertension

A total of 1697 patients were included in the study. The sociodemographic and clinical characteristics are shown in Table 1. The average age of the patients was 71.04 ± 8.56 years old. The majority of the patients were female (1028, 60.6%), married (1643, 86.2%), living with others (1560, 92%), had a high school or below educational level (1157, 68.2%), had a monthly income below 5000 China yuan (1267, 74.7%), had more than one complication (1327, 78.2%) and took more than one medication (66.2%). Young age (*P* < 0.001), married status (*P* = 0.004), and high education level (*P* < 0.001) were significantly associated with a high degree of medication adherence. A high degree of medication adherence was associated with high social support (*P* < 0.001) and high health literacy (*P* < 0.001). Interestingly, a high degree of medication adherence was significantly related to frequent exercise and vegetable consumption (all *P* < 0.001), as shown Additional file 1: Table S1. Although the control rate of blood pressure was not correlated with the levels of medication adherence in the study, comorbidities including diabetes and cardiovascular and emergency events were more frequently observed in patients with a high degree of medication adherence (Additional file 1: Table S1).

**Table 1:** Characteristics of 1697 participants stratified by medication adherence levels

Variables	Total (n = 1697)	Medication adherence			*P* value
		Low (n = 654)	Medium (n = 704)	High (n = 339)	
Age (years), Mean ± SD	71.04 ± 8.56	72.95 ± 8.79	70.20 ± 8.15	69.08 ± 8.23	** < 0.001**
*Sex, n (%)*					0.607
Male	669 (39.4)	255 (39.0)	275 (39.1)	139 (41.0)	
Female	1028 (60.6)	399 (61.0)	429 (60.9)	200 (59.0)	
*Marital, n (%)*					**0.004**
Married	1463 (86.2)	538 (82.3)	631 (89.6)	294 (86.7)	
Single, divorced or other	234 (13.8)	116 (17.7)	73 (10.4)	45 (13.3)	
*Living status, n (%)*					0.078
With others	1560 (91.9)	589 (90.1)	658 (93.5)	313 (92.3)	
Alone	137 (8.1)	65 (9.9)	46 (6.5)	26 (7.7)	
*Education level, n (%)*					** < 0.001**
Less than high school	249 (14.7)	141 (21.6)	78 (12.1)	30 (8.8)	
High school	908 (53.5)	343 (52.4)	366 (56.9)	199 (58.7)	
Some college and beyond	540 (31.8)	170 (26.0)	199 (30.9)	110 (32.4)	
*Income (China Yuan), n (%)*					0.346
< 5000	1267 (74.7)	498 (76.1)	518 (73.6)	251 (74.0)	
≥ 5000	430 (25.3)	156 (23.9)	186 (26.4)	88 (26.0)	
*Duration of hypertension, n (%)*					0.140
2–5 years	358 (22.5)	160 (24.5)	209 (29.7)	98 (28.9)	
5–10 years	619 (39.0)	243 (37.2)	254 (36.1)	122 (36.0)	
≥ 10 years	611 (38.5)	251 (38.4)	241 (34.2)	119 (35.1)	
*Comorbidity, n (%)*					**0.001**
1	370 (21.8)	117 (17.9)	172 (24.4)	81 (23.9)	
2	619 (36.5)	229 (35.0)	271 (38.5)	119 (35.1)	
3	463 (27.3)	184 (28.1)	183 (26.0)	96 (28.3)	
≥ 4	245 (14.4)	124 (19.0)	78 (11.1)	43 (12.7)	
*Medication, n (%)*					** < 0.001**
1	573 (33.8)	179 (27.4)	264 (37.5)	130 (38.3)	
2	576 (33.9)	237 (36.2)	249 (35.4)	90 (26.5)	
≥ 3	548 (32.3)	238 (36.4)	191 (27.1)	119 (35.1)	
Social support, Mean ± SD	34.88 ± 6.54	33.19 ± 6.21	35.67 ± 6.12	36.53 ± 7.24	** < 0.001**
Objective support, Mean ± SD	8.14 ± 2.31	8.02 ± 2.43	8.16 ± 2.19	8.31 ± 2.31	0.153
Subjective support, Mean ± SD	19.07 ± 3.96	17.99 ± 3.57	19.51 ± 3.98	20.20 ± 4.11	** < 0.001**
Support utilization, Mean ± SD	7.68 ± 2.15	7.18 ± 3.02	7.99 ± 2.10	8.02 ± 2.34	** < 0.001**
Health literacy, Mean ± SD	105.32 ± 16.37	98.91 ± 18.30	109.68 ± 13.07	108.63 ± 14.57	** < 0.001**
Information acquisition ability, Mean ± SD	39.63 ± 6.83	36.99 ± 7.90	41.35 ± 5.33	41.12 ± 5.68	** < 0.001**
Communication interaction ability, Mean ± SD	39.07 ± 6.59	36.68 ± 7.36	40.63 ± 5.52	40.42 ± 5.62	** < 0.001**
Health improvement willingness, Mean ± SD	17.53 ± 3.01	16.45 ± 3.32	18.31 ± 2.46	17.97 ± 2.81	** < 0.001**
Economic support willingness, Mean ± SD	9.10 ± 1.35	8.78 ± 1.48	9.40 ± 1.10	9.12 ± 1.41	** < 0.001**

A *p*-value of less than 0.05 was regarded as significant and marked as Bold

### Correlations between study variables

The correlations among medication adherence, social support, health literacy, age, education level, and marital status are shown in Table 2. Social support (r = 0.212, *P* < 0.001), health literacy (r = 0.330, *P* < 0.001), education level (r = 0.118, *P* < 0.001) and marital status (r = 0.085, *P* < 0.001) were positively correlated with medication adherence, while age was negatively associated with medication adherence (r = −0.155, *P* < 0.001). Social support was positively correlated with health literacy (r = 0.364, *P* < 0.001), education level (r = 0.231, *P* < 0.001) and marital status (r = 0.320, *P* < 0.001) but negatively associated with age (r = −0.238, *P* < 0.001). Health literacy was positively associated with education level (r = 0.294, *P* < 0.001) and marital status (r = 0.208, *P* < 0.001) but negatively associated with age (r = −0.391, *P* < 0.001).

**Table 2:** Spearman's correlation matrix of the study variables

	Age	Marital	Education level	Social support	Health literacy	Medication adherence
1. Age	1					
2.Marital	−0.246^***^	1				
3. Education level	−0.357^***^	0.226^***^	1			
4. Social support	−0.238^***^	0.320^***^	0.231^***^	1		
5. Healthy literacy	−0.391^***^	0.208^***^	0.294^***^	0.364^***^	1	
6. Medication adherence	−0.188^***^	0.085^***^	0.118^***^	0.212^***^	0.330^***^	1

^***^ indicates a *p*-value of less than 0.001

### Test of study models

**Fig. 2:**
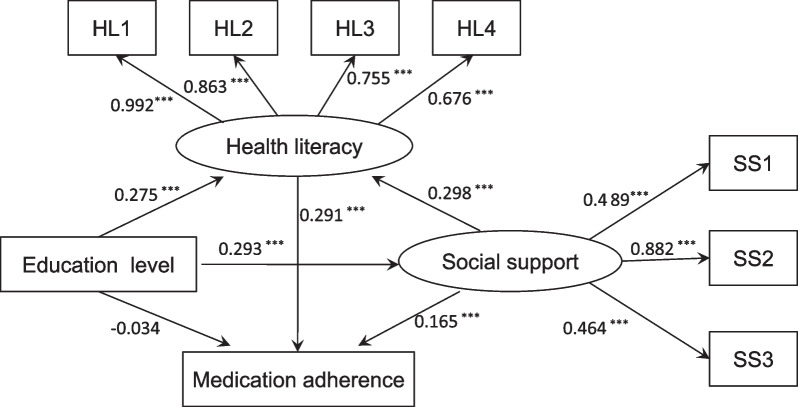
The final model (model 1) and standardized model paths for medication adherence in patients with hypertension. SS1 represents the dimension of objective support. SS2 represents the dimension of subjective support. SS3 represents the dimension of support utilization. HL1 represents the dimension of information acquisition ability. HL2 represents the dimension of communication interaction ability. HL3 represents the dimension of health improvement willingness. HL4 represents the dimension of economic support willingness. Circles indicate latent variables. Rectangles indicate exogenous variables. All path coefficients were standardized. Statistical significance: ***P < 0.001; **P < 0.01; *P < 0.05

**Fig. 3:**
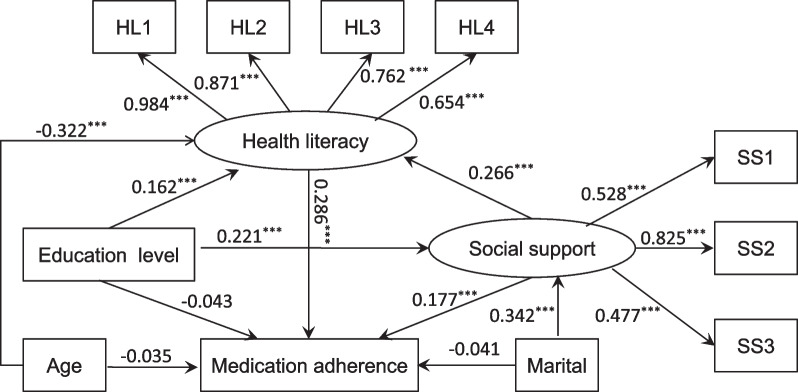
The final model (model 2) and standardized model paths for medication adherence in the patients with hypertension. SS1 represents the dimension of objective support. SS2 represents the dimension of subjective support. SS3 represents the dimension of support utilization. HL1 represents the dimension of information acquisition ability. HL2 represents the dimension of communication interaction ability. HL3 represents the dimension of health improvement willingness. HL4 represents the dimension of economic support willingness. Circles indicate latent variables. Rectangles indicate exogenous variables. All path coefficients were standardized. Age and marital status were controlled. Statistical significance: ***P < 0.001; **P < 0.01; *P < 0.05

The structural model in this study contained two observed variables (medication adherence and education level) and two latent variables (social support and health literacy). Figure 2 (model 1) illustrates the results of the SEM used to explore the interactions among the variables. Standardized direct, indirect, and total effects are shown in Table 3. Health literacy had a direct effect on medication adherence (*β* = 0.291, *P* < 0.001). Social support had a direct effect on medication adherence (*β* = 0.165, *P* < 0.001) and health literacy (*β* = 0.298, *P* < 0.001). While education level had no direct influence on medication adherence (*β* = −0.034, *P* = 0.170), it had an indirect effect on adherence through social support (*β* = 0.048, *P* < 0.001) and health literacy (*β* = 0.080, *P* < 0.001), as shown Table 3. Additionally, social support and health literacy had sequential mediating effects on the relationship between education level and medication adherence (*β* = 0.025, *P* < 0.001). Social support also indirectly affected medication adherence via health literacy (*β* = 0.087, *P* < 0.001). Furthermore, education level influenced health literacy directly (*β* = 0.275, *P* < 0.001) and indirectly through social support (*β* = 0.087, *P* < 0.001) and directly affected social support (*β* = 0.293, *P* < 0.001). It is worth noting that social support and health literacy fully mediated the association between education level and medication adherence. The model fit (model 1) was confirmed to be acceptable (IFI = 0.986, CFI = 0.986, NFI = 0.983, RMSEA = 0.059). After controlling the variables for age and marital status, we obtained similar results for the 4 variables (medication adherence, social support, health literacy and education), as shown Fig. 3 (model 2) and Table 4. We also observed that both age (*β* = −0.035, *P* = 0.213) and marital status (*β* = −0.041, *P* = 0.122) had no direct influence on medication adherence but indirectly affected medication adherence through health literacy (*β* = −0.322, *P* < 0.001) and social support (*β* = 0.342, *P* < 0.001), respectively. Model 2 was also confirmed to have an acceptable fit (IFI = 0.963, CFI = 0.963, NFI = 0.959, RMSEA = 0.074).

**Table 3:** Direct, indirect, and total effects of the study variables in model 1

	β	SE	Percentile 95% CI		Bias-corrected percentile 95% CI		*P value*
			Lower	Upper	Lower	Upper	
*Standardized direct effect*							
Social support → Medication adherence	0.165	0.028	0.110	0.220	0.106	0.228	< 0.001
Health literacy → Medication adherence	0.291	0.032	0.228	0.355	0.224	0.366	< 0.001
Education level → Medication adherence	−0.034	0.026	−0.085	0.016	−0.082	0.015	0.170
Social support → Health literacy	0.298	0.032	0.236	0.360	0.243	0.367	< 0.001
Education level → Health literacy	0.275	0.026	0.225	0.326	0.219	0.329	< 0.001
Education level → Social support	0.293	0.024	0.245	0.341	0.235	0.357	< 0.001
*Standardized indirect effect*							
Education level → Social support → Medication adherence	0.048	0.009	0.030	0.067	0.031	0.069	< 0.001
Education level → Health literacy → Medication adherence	0.080	0.012	0.056	0.104	0.057	0.107	< 0.001
Education level → Social support → Health literacy → Medication adherence	0.025	0.005	0.016	0.035	0.017	0.037	< 0.001
Social support → Health literacy → Medication adherence	0.087	0.014	0.060	0.114	0.062	0.117	< 0.001
Education level → Social support → Health literacy	0.087	0.013	0.063	0.112	0.064	0.115	< 0.001
*Standardized total effect*							
Education level → Medication adherence	0.119	0.024	0.073	0.166	0.073	0.166	< 0.001
Social support → Medication adherence	0.252	0.028	0.196	0.307	0.190	0.319	< 0.001
Education level → Health literacy	0.363	0.023	0.318	0.408	0.311	0.414	< 0.001

Standardized total effect refers to standardized direct effect plus standardized indirect effect

**Table 4:** Direct, indirect, and total effects of the study variables in model 2

	β	SE	Percentile 95% CI		Bias-corrected percentile 95% CI		*P value*
			Lower	Upper	Lower	Upper	
*Standardized direct effect*							
Social support → Medication adherence	0.177	0.032	0.114	0.240	0.109	0.248	< 0.001
Health literacy → Medication adherence	0.286	0.034	0.220	0.352	0.217	0.360	< 0.001
Education level → Medication adherence	−0.043	0.026	−0.094	0.009	−0.095	0.010	0.106
Social support → Health literacy	0.266	0.030	0.208	0.325	0.210	0.333	< 0.001
Education level → Health literacy	0.162	0.027	0.110	0.215	0.105	0.215	< 0.001
Education level → Social support	0.221	0.026	0.170	0.272	0.167	0.277	< 0.001
Age → Medication adherence	−0.035	0.028	−0.090	0.020	−0.085	0.020	0.213
Marital → Medication adherence	−0.041	0.027	−0.094	0.011	−0.093	0.012	0.122
Age → Health literacy	−0.322	0.029	−0.377	−0.266	−0.380	−0.264	< 0.001
Marital → Social support	0.342	0.029	0.285	0.398	0.267	0.422	< 0.001
*Standardized indirect effect*							
Education level → Social support → Medication adherence	0.039	0.009	0.022	0.056	0.024	0.059	< 0.001
Education level → Health literacy → Medication adherence	0.046	0.010	0.027	0.066	0.028	0.068	< 0.001
Education level → Social support → Health literacy → Medication adherence	0.017	0.003	0.010	0.024	0.012	0.026	< 0.001
Social support → Health literacy → Medication adherence	0.076	0.012	0.052	0.100	0.054	0.105	< 0.001
Education level → Social support → Health literacy	0.059	0.010	0.039	0.078	0.042	0.081	< 0.001
Age → Health literacy → Medication adherence	−0.092	0.014	−0.118	−0.065	−0.124	−0.070	< 0.001
Marital → Social support → Medication adherence	0.060	0.012	0.036	0.085	0.038	0.086	< 0.001
Marital → Social support → Health literacy → Medication adherence	0.026	0.005	0.016	0.036	0.018	0.038	< 0.001
*Standardized total effect*							
Education level → Medication adherence	0.060	0.026	0.009	0.110	0.010	0.113	0.022
Social support → Medication adherence	0.253	0.032	0.191	0.315	0.183	0.328	< 0.001
Education level → Health literacy	0.221	0.026	0.171	0.271	0.168	0.273	< 0.001
Marital → Medication adherence	0.045	0.026	−0.006	0.096	−0.004	0.098	< 0.001
Age → Medication adherence	−0.133	0.030	−0.193	−0.074	−0.290	0.024	< 0.001

Standardized total effect refers to standardized direct effect plus standardized indirect effect

## Discussion

In this study, the proportion of participants with a medication adherence score of < 6 was 38.5%, showing an inadequate degree of medication adherence, despite the prevalence rates being lower than previous reports (45.2% to 78.7%) [28–30]. There is an urgent need to improve medication adherence in the population. Most importantly, we employed SEM to clarify the impact of factors including age, education level, social support and health literacy on medication adherence among hypertensive patients. The results show that social support and health literacy not only directly affected the degree of medication adherence among patients with hypertension but also indirectly mediated the associations of medication adherence with age, education level, and marital status. Therefore, it is very important to consider an individual's levels of social support and health literacy when working to improve medication adherence [31].

Adequate social support can be obtained from families, friends, peers, health care professionals and organizations, even from internet, which has been shown to improve medication adherence and the quality of patients' lives [32–34]. Herein, we found that that social support was directly associated with medication adherence, which is similar to the results from previous studies [9–11]. However, the mechanisms of the association between social support and medication adherence have yet to be fully elucidated. When patients with hypertension find it easy to obtain adequate levels of family support, his or her spouses or other caregivers provide practical support by directly helping and supervising their medication [9, 10]. Patients receiving support from family members and/or friends may feel a greater sense of self-worth [35], which can encourage optimism regarding treatment. Higher social support is associated with better psychological health, meaning that the improvement of medication adherence may be induced by good emotion [36]. Social support is also multifaceted and may help patients remain active in their care when faced with physical, social and economic vulnerabilities [37]. The practical support of a social network may be another explanation of the effect of social support on medication adherence.

A barrier to hypertension self-management is health literacy [38, 39], defined as the "capacity to obtain, process, and understand basic health information and services needed to make appropriate health decisions". Limited health literacy is known to contribute to poor medication adherence among patients with chronic diseases [40], including hypertension [41, 42], thus resulting in poor management of hypertension and poor blood pressure control. Health literacy has also been positively associated with social support among patients with chronic kidney disease and patients with coronary heart disease. In our study, health literacy was not only associated with medication adherence but also identified as a mediator of the relationship between social support and medication adherence, indicating that health literacy may serve as a core determinant of medication adherence among patients with hypertension. Improving health literacy has been suggested as a possible intervention target to increase medication adherence [41, 43] as well as other hypertension self-care behaviors [42, 44]. Although health literacy has a positive impact on medication adherence in patients with hypertension, little is known about the mechanism of this relationship. Four core elements of health literacy include knowledge, attitude, skill and behavior, and each domain is essential and critical for processing medication information and correct medication use. People with low levels of health literacy were more likely to misinterpret information on drug labels [45], resulting in low medication adherence due to adverse events. Low health literacy is associated with reduced patient-centered communication and, in turn, diminished shared decision-making [46]. Moreover, a recent study has indicated that self-efficacy may partially mediate the effect of health literacy on medication adherence in a hypertensive population [28]. In fact, patients with high levels of self-efficacy had greater confidence that they would be willing to take drugs as prescribed on different occasions [47].

As in previous studies, we also observed that many sociodemographic factors, including age, education level, and marital status, were significantly associated with medication adherence among patients with hypertension in Shanghai, China. However, how the factors influence the levels of medication adherence in the hypertensive population remains unclear. Our research demonstrated that the factors of age, educational level and marital status cannot directly affect the levels of medication adherence of patients with hypertension but indirectly influence medication adherence through the mediating role of health literacy and/or social support, confirming them as the critical determinants for medication adherence in the hypertension population. Among lifestyle factors including smoking, drinking, exercise and vegetable usage, regular exercise is significantly positively correlated with medication adherence, which is consistent with the previous findings indicating that exercise adherence and medication adherence have the same impact factors. Interestingly, we observed that the frequency of vegetable usage was negatively associated with medication adherence in the present study, which is contrary to a previous study in Jordan [48]. The possible causes involved a high proportion (58.1%) of patients with diabetes in our study, less than 33.2% in Jordan's study, most of whom were worried about sugar intake, as the fruit and/or vegetables were not chosen correctly. The factors of sex and duration of hypertension were not associated with medication adherence in the study, and the possible cause may be involved in different populations and comorbidities. Economic income was also not associated with medication adherence in our study; however, it was significantly correlated with medication adherence in other studies. The causes for the difference may be sufficient economic support for medication consumption in Shanghai even if there is a low level of income. The number of comorbidities and the number of medications taken were significantly associated with medication adherence in our study, the results of which were similar to a large number of previous studies [49, 50].

## Strengths and limitations

The study clarified the relationship among medication adherence, education level, social support, and health literacy and provided meaningful information for health providers of patients. The current study has several potential limitations. Therefore, in this cross-sectional study, it was not possible to determine the temporality sequence between medication adherence and social support and health literacy. The nature of a cross-sectional study also limits our ability to make causal inferences regarding whether social support and health literacy casually influence medication adherence. Since the information of social support, health literacy and medication adherence were all self-reported and collected through questionnaires, it is possible that misclassification and overestimation may have occurred.

## Conclusion

The prevalence rate of medication nonadherence among patients with hypertension was quite high in our cohort (38%), although it was lower than the rates reported in previous studies. The main determinants of nonadherence include education level, social support and health literacy, among which complex interactions exist according to SEM analysis. The assessment of social support, health literacy and other factors can help to elucidate variations in medication adherence across different hypertension populations. Improving the social support and health literacy of patients with hypertension can positively influence their medication adherence and therapeutic outcomes. This research is also helpful for the government to make some health policies to improve medication adherence in hypertension.

## Supplementary Information

**Additional file 1.** Table S1. Lifestyle and clinical variables of 1697 participants.

## Data Availability

The datasets generated and analyzed during the current study are not publicly available owing to data security issues, but are available from the corresponding author on reasonable request.

## Abbreviations

BMI: Body mass index
HbA1c: Hemoglobin A1c
MMAS-8: Morisky medication adherence scale
SSRS: Social support rate scale
HeLMS: Health literacy management scale
SDs: Standard deviations
ANOVA: Analysis of variance
SEM: Structural equation model
CI 95%: Confidence interval CI
IFI: Incremental fit index
CFI: Comparative fit index
NFI: Normed fit index
RMSEA: Root mean squared error of approximation
