# Supplementary material for: Impact of health literacy and social support on medication adherence in patients with hypertension: a cross-sectional community-based study
Source: BMC Cardiovasc Disord. 2023 Feb 19;23:93. doi: 10.1186/s12872-023-03117-x (PMC9940429; doi:10.1186/s12872-023-03117-x)
Supplement: Supplementary file 1 — Additional file 1. Table S1. Lifestyle and clinical variables of 1697 participants. [file 12872_2023_3117_MOESM1_ESM.docx]

**Supplementary Table 1. Lifestyle and clinical variables of 1697 participants stratified by medication adherence level**

| **Variables** | **Total (n=1697)** | **Medication Adherence** | | | **P value** |
| --- | --- | --- | --- | --- | --- |
|  |  | **Low (n=654)** | **Medium (n=704)** | **High (n=339)** |  |
| **BMI, n (%)** |  |  |  |  | 0.353 |
| < 25 | 1011 (62.8) | 400 (64.0) | 415 (63.3) | 196 (59.4) |  |
| ≥ 25 | 600 (37.2) | 225 (36.0) | 241 (36.7) | 134 (40.6) |  |
| **Smoking, n (%)** |  |  |  |  | 0.993 |
| Yes | 141 (8.3) | 55 (8.4) | 58 (8.2) | 28 (8.3) |  |
| No | 1556 (91.7) | 599 (91.6) | 646 (91.8) | 311 (91.7) |  |
| **Drinking, n (%)** |  |  |  |  | 0.291 |
| Yes | 75 (4.4) | 28 (4.3) | 41 (5.8) | 6 (1.8) |  |
| No | 1622 (95.6) | 626 (95.7) | 663 (94.2) | 333 (98.2) |  |
| **Exercise, n (%)** |  |  |  |  | **< 0.001** |
| 1 | 529 (31.2) | 267 (40.8) | 185 (26.3) | 77 (22.7) |  |
| 2 | 632 (37.2) | 216 (33.0) | 283 (40.2) | 133 (39.2) |  |
| 3 | 536 (31.6) | 171 (26.1) | 236 (33.5) | 129 (38.1) |  |
| **Vegetable, n (%)** |  |  |  |  | **< 0.001** |
| 2 | 246 (14.5) | 86 (13.1) | 100 (14.2) | 60 (17.7) |  |
| 3 | 740 (43.6) | 255 (39.0) | 325 (46.2) | 160 (47.2) |  |
| 4 | 711 (41.9) | 313 (47.9) | 279 (39.6) | 119 (35.1) |  |
| **Systolic blood pressure, n (%)** |  |  |  |  | 0.120 |
| < 140 | 903 (54.9) | 361 (56.7) | 350 (51.9) | 192 (57.5) |  |
| ≥ 140 | 743 (45.1) | 276 (43.3) | 325 (48.1) | 142 (42.5) |  |
| **Diastolic blood pressure, n (%)** |  |  |  |  | 0.103 |
| < 90 | 1439 (87.5) | 572 (89.8) | 575 (85.4) | 292 (87.4) |  |
| ≥ 90 | 205 (12.5) | 65 (10.2) | 98 (14.6) | 42 (12.6) |  |
| **Blood Glucose, n (%)** |  |  |  |  | 0.264 |
| < 6.1 | 829 (52.2) | 302 (49.7) | 348 (53.5) | 179 (54.4) |  |
| ≥ 6.1 | 758 (47.8) | 306 (50.3) | 302 (46.5) | 150 (45.6) |  |
| **HbA1c, n (%)** |  |  |  |  | 0.530 |
| < 6.5 | 999 (65.5) | 361 (62.7) | 437 (69.8) | 201 (62.2) |  |
| ≥ 6.5 | 526 (34.5) | 215 (37.3) | 189 (30.2) | 122 (37.8) |  |
| **TG, n (%)** |  |  |  |  | 0.934 |
| < 1.7 | 1008 (66.0) | 379 (65.5) | 416 (66.2) | 213 (66.6) |  |
| ≥ 1.7 | 519 (34.0) | 200 (34.5) | 212 (33.8) | 107 (33.4) |  |
| **TC, n (%)** |  |  |  |  | 0.184 |
| < 5.17 | 862 (56.5) | 323 (55.8) | 370 (58.9) | 169 (52.8) |  |
| ≥ 5.17 | 665 (43.5) | 256 (44.2) | 258 (41.1) | 151 (47.2) |  |
| **LDL, n (%)** |  |  |  |  | 0.831 |
| < 3.4 | 1202 (78.8) | 453 (78.5) | 493 (78.4) | 256 (80.0) |  |
| ≥ 3.4 | 324 (21.2) | 124 (21.5) | 136 (21.6) | 64 (20.0) |  |
| **Diabetes, n (%)** |  |  |  |  | **0.001** |
| Yes | 986 (58.1) | 421 (64.4) | 374 (53.1) | 191 (56.3) |  |
| No | 711 (41.9) | 233 (35.6) | 330 (46.9) | 148 (43.7) |  |
| **Hyperlipidemia, n (%)** |  |  |  |  | 0.904 |
| Yes | 722 (42.5) | 275 (42.0) | 304 (43.2) | 143 (42.2) |  |
| No | 975 (57.5) | 379 (58.0) | 400 (56.8) | 196 (57.8) |  |
| **CVD, n (%)** |  |  |  |  | 0.867 |
| Yes | 289 (17.0) | 121 (18.5) | 100 (14.2) | 68 (20.1) |  |
| No | 1408 (83.0) | 533 (81.5) | 604 (85.8) | 271 (79.9) |  |
| **Stroke, n (%)** |  |  |  |  | **< 0.001** |
| Yes | 145 (8.5) | 81 (12.4) | 42 (6.0) | 22 (6.5) |  |
| No | 1552 (91.5) | 573 (87.6) | 662 (94.0) | 317 (93.5) |  |
| **Event, n (%)** |  |  |  |  | **< 0.001** |
| 1 | 1209 (71.2) | 447 (68.3) | 483 (68.6) | 279 (82.3) |  |
| ≥2 | 488 (28.8) | 207 (31.7) | 221 (31.4) | 60 (17.7) |  |

BMI, body mass index; TG, triglyceride; TC, total cholesterol; LDL, low-density lipoprotein; CVD, cardiovascular disease.
